# Unconventional Management of Partial Placenta Accreta for Uterine Preservation: A Case Report

**DOI:** 10.7759/cureus.93867

**Published:** 2025-10-05

**Authors:** Melody Salimian, Ju Yong Koh

**Affiliations:** 1 Obstetrics and Gynecology, Midwestern University Chicago College of Osteopathic Medicine, Downers Grove, USA; 2 Obstetrics and Gynecology, Northwestern Medicine, Geneva, USA

**Keywords:** dilation and curettage, hemolysis elevated liver enzymes and low platelet count, hysterectomy, placenta accreta spectrum (pas), retained products of conception (rpoc)

## Abstract

We present the case of a 35-year-old G2P1102 who underwent a unique approach to managing partial placenta accreta for uterine preservation. The patient was diagnosed with atypical hemolysis, elevated liver enzymes, and low platelet count (HELLP) syndrome and subsequently underwent a repeat cesarean delivery at 36 weeks of gestation. Intraoperatively, approximately 5% of the placenta was determined to remain adherent to the uterine cavity. Initial attempts at complete placental removal were unsuccessful. After hemostasis was obtained, it was decided that the patient would undergo expectant management with close observation. At the six-week postpartum appointment, retained products of conception were suspected, contributing to ongoing uterine bleeding. Hysteroscopic tissue resection and dilation, and curettage were used to remove remnant placental tissues, then the Mirena intrauterine device (IUD) was inserted at the end. The procedure was successful, preserving the uterus and resolving the patient's bleeding. This case highlights a potential alternative approach to managing partial placenta accreta. Further research is needed to evaluate the efficacy and safety of this method in broader clinical practice.

## Introduction

Placenta accreta is a critical obstetrical condition in which abnormal trophoblastic invasion causes the placenta to attach firmly to the myometrium, preventing normal placental separation after delivery [1]. The condition falls under placenta accreta spectrum (PAS), which encompasses three variations: placenta accreta, where the placenta adheres superficially to the myometrium; placenta increta, where it invades the myometrium; and placenta percreta, where it penetrates through the myometrium and may affect surrounding organs. Normal placental development relies on a delicate balance of processes, including trophoblast invasion, maternal tissue reorganization, and proper spiral artery formation, all of which are tightly regulated through complex signaling pathways and immune interactions [2]. When the uterine lining has been previously injured, this balance can be compromised, leading to impaired communication between regulatory pathways and disruption of normal vascular and tissue adaptations, leading to PAS [2]. A 2016 study reported a placenta accreta prevalence of just under four per 1,000 deliveries [3]. Risk factors for PAS include advanced maternal age, previous uterine procedures such as dilation and curettage, and repeat cesarean sections [1]. The global surge in cesarean deliveries has made prior cesarean section with an anterior low-lying placenta or placenta previa the leading risk factor, accounting for over 90% of PAS cases [4]. A 10-year population study in Australia found that PAS significantly elevates maternal mortality and has been associated with an 18-fold increase in maternal morbidity, often leading to severe complications such as massive hemorrhage, intensive care unit (ICU) admissions, and hysterectomy [5,6].

The International Federation of Gynecology and Obstetrics has outlined several methods for managing PAS, ranging from leaving the placenta in situ to manual placenta removal and surgical approaches involving resection and pelvic devascularization [7]. However, there are currently no clear guidelines on conservative management strategies for PAS. In a recent systematic review of 14 international and national guidelines, no consistent approach to conservative management was identified [8]. One of the more commonly described options is to leave the placenta in situ; however, there is no consensus on standardized recommendations for monitoring or follow-up [8]. This gap highlights the need for further research into conservative management strategies and the development of standardized protocols to guide clinical practice and potential interventions.

## Case presentation

We report the case of a 35-year-old G2P1102 who underwent unconventional management of partial placenta accreta for uterine preservation. The patient has a past medical history of oligohydramnios antepartum and a previous cesarean delivery. The patient presented at 33w1d with vague right upper quadrant abdominal pain and diarrhea. Laboratory studies at that time showed mild elevations in liver enzymes, slight thrombocytopenia, and increased urine protein-to-creatinine ratio (UPCR) (Table 1).

**Table 1:** Laboratory values and qualitative interpretation during the patient’s pregnancy course. Values are shown with reference ranges, actual numerical values, and qualitative interpretation. ALT, alanine aminotransferase; AST, aspartate aminotransferase; UPCR, urine protein-to-creatinine ratio

Parameter	Gestational age: 33w1d	Gestational age: 36w5d	Reference range	Interpretation
ALT	48	63	7-56 U/L	Increased
AST	40	42	10-40 U/L	Increased
Platelets	140	119	150-400 K/μL	Decreased
UPCR	0.73	0.91	<0.3	Increased

The patient’s abdominal pain quickly resolved, and with normotensive blood pressures, she was allowed to progress further in her pregnancy course with close monitoring. At 36w5d, repeat labs demonstrated further liver enzyme elevations, decreased platelets, and higher UPCR (Table 1). The patient continued to be normotensive, and further lab work ensued to determine the etiology of the abnormal lab results. At 36w6d, the hepatitis panel and coagulopathy panel came back negative, and given the overall clinical scenario, an atypical presentation of HELLP syndrome was diagnosed. Per recommendation from the maternal fetal medicine team, repeat low transverse cesarean delivery was performed to deliver a healthy baby boy without complication. The delivery of the placenta, which was located posteriorly, was complicated by the severe adherence of roughly 5% of the remaining tissue to the posterior wall of the uterus. Several attempts to remove this remaining tissue manually were unsuccessful. There was no known finding of PAS during any antepartum sonograms.

Postpartum hemorrhage was controlled, and excellent hemostasis was achieved with uterotonics along with placement of a Bakri balloon for the uterine tamponade effect. Given the patient’s quantitative blood loss of 1873 ml during surgery and a vasovagal episode in the post-anesthesia care unit, the decision was made to administer one unit each of packed red blood cells, cryoprecipitate, and fresh frozen plasma. At that time comprehensive blood count was within normal limits, including a white blood count of 10.5. The patient was started on Ancef two grams, to be continued every eight hours until removal of the Bakri balloon at the 24-hour timepoint. She was also started on magnesium sulfate and pitocin for 24 hours postpartum. A total of 100 cc of blood loss was collected from the Bakri device. Fundal massage continued to demonstrate a firm uterus, and no vaginal bleeding was noted around the Bakri device. No other significant events occurred during the patient’s hospital stay. On postoperative day three, the patient was clinically stable and was discharged home.

**Figure 1:**
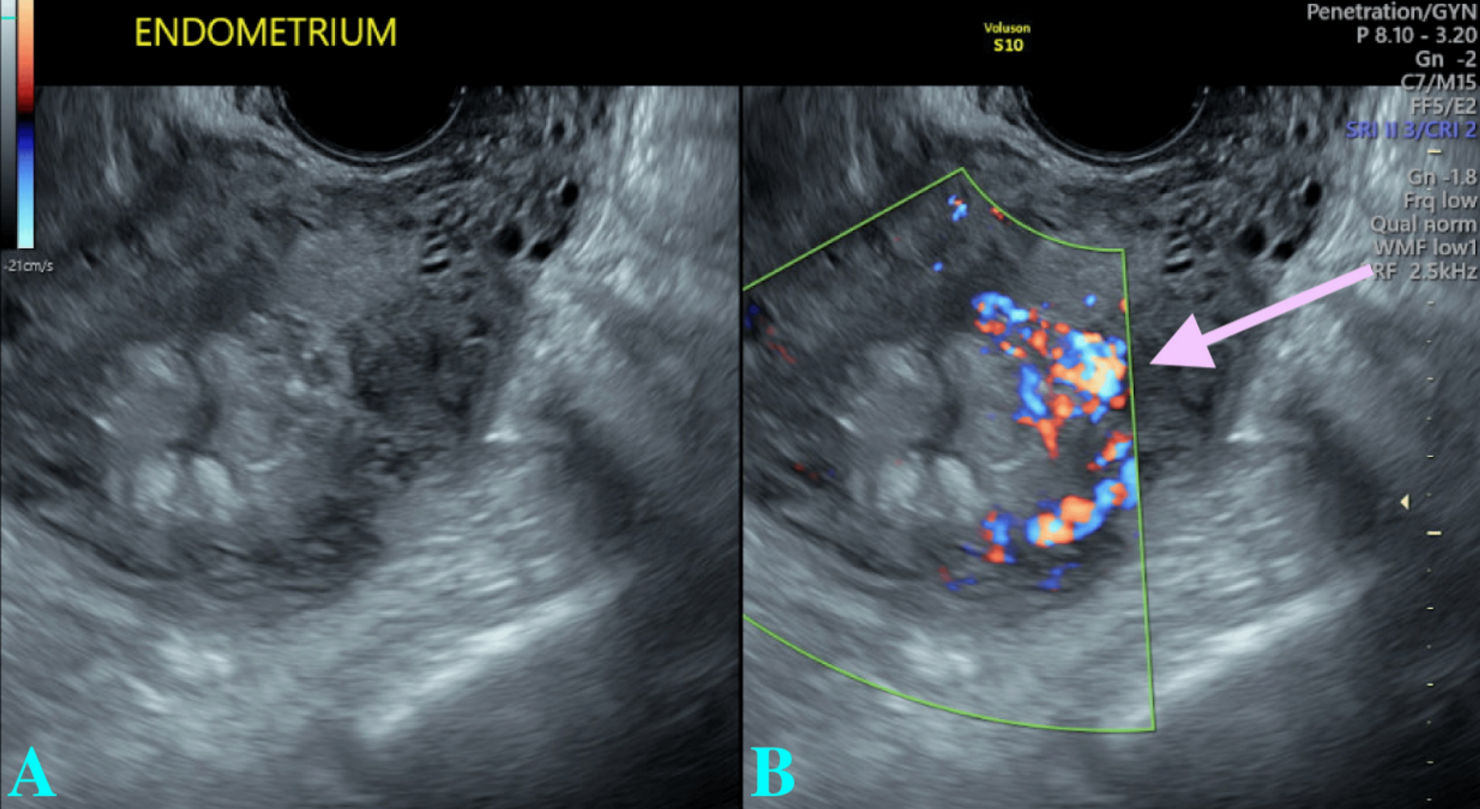
Ultrasound of the endometrium. (A) Endometrium before color Doppler flow. (B) Endometrium after color Doppler flow. The arrow indicates suspected retained products of conception with internal blood flow.

At the six-week postpartum visit, the patient endorsed occasional passing of clots and moderately heavy vaginal bleeding. Office exam noted a small amount of uterine bleeding. Pelvic ultrasound revealed 36 mm of endometrial thickness with heterogeneous retained products within the endometrial cavity, with internal vascularity (Figures 1A, 1B).

**Figure 2:**
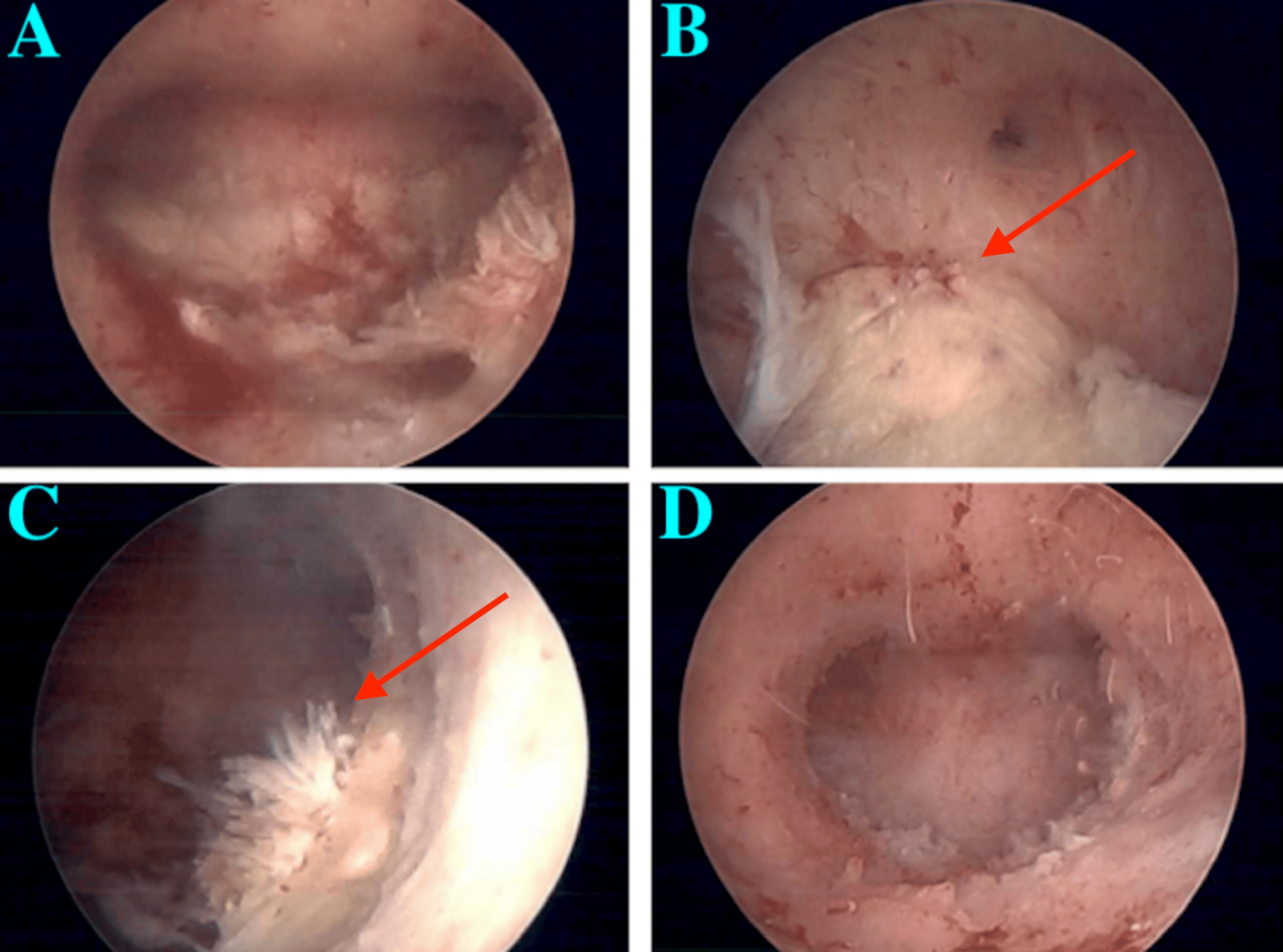
Hysteroscopic images of the uterus. (A) Uterine cavity before curettage. (B) Arrow indicating suspected retained products of conception. (C) Arrow indicating another uterine area with suspected retained products of conception. (D) Uterine cavity after curettage.

The patient was scheduled for a hysteroscopy, dilation, and curettage with the Myosure device, a hysteroscopic instrument designed for the removal of intrauterine pathology such as retained products of conception, and agreed to a Mirena IUD placement. Before the procedure, the patient was placed on a daily oral progestin regimen for additional bleeding control with minimal improvement. During surgery, hysteroscopic resection was performed to remove the remaining placental tissue (Figures 2A-2C).

By the end of the procedure, the uterine cavity was smooth, uniform, and all remaining placenta had been removed (Figure 2D). The Mirena IUD was successfully placed in alignment with the patient's choice of contraception, offering added benefits of bleeding control and endometrial thinning. Biopsy results of the intrauterine lesions revealed chronic endometritis and multiple fragments of degenerated/devitalized tissue. While a conclusive interpretation of the origin of the devitalized tissue was not possible, it was suspected to represent degenerated placental tissue, particularly given the clinical context. At the two-week post-operative visit, the patient was doing well, with no bleeding concerns, and expressed satisfaction with the results of the procedure. 

## Discussion

Various management strategies have been employed for the management of PAS. The preferred approach to PAS has typically been to proceed with cesarean hysterectomy [1]. Suspected placenta accreta can often be identified through ultrasound, allowing for early management planning and discussions with the patient during the antenatal period. Although in cases like ours, placenta accreta could be identified incidentally in the OR, requiring immediate clinical decisions to be made for either cesarean hysterectomy or an attempt at uterine preservation. The risks versus benefits of both these management strategies have long been studied.

In a retrospective cohort study conducted at two tertiary care centers in Utah between 1996 and 2008, 57 cases with patients having suspected placenta accreta were examined [9]. Of the cohort, 56 patients ultimately required hysterectomy due to placental adherence. In 15 cases, an initial attempt at manual placental removal resulted in uncontrolled hemorrhage, necessitating hysterectomy [9]. Although the study did not specify which interventions were implemented to control hemorrhage, the findings strongly suggested that proceeding directly to hysterectomy without attempting placental removal significantly reduced maternal morbidity [9]. In recent years, several studies have investigated other uterine conservation techniques to manage PAS. A recent meta-analysis involving 16 studies and 2,300 patients examined outcomes between patients who underwent cesarean hysterectomy and those managed with conservative approaches, such as local placental resection or leaving the placenta in situ [10]. The study aimed to assess differences in maternal morbidity, blood loss, and overall clinical outcomes between these management strategies. The cesarean hysterectomy group experienced greater blood loss than both the local resection and placenta in situ groups [10]. Management of PAS with cesarean hysterectomy also resulted in a greater need for blood transfusion intraoperatively compared to the local resection group. Additionally, the cesarean hysterectomy cohort had more ICU admissions than the expectant management placenta in situ cohort [10]. These findings demonstrated that conservative measures could be found to be equally, if not more, effective methods of reducing morbidity among patients with PAS [10]. In another literature review analyzing 1,918 PAS cases from 12 studies, it was found that 27.1% of cases were managed by leaving the placenta in situ [11]. Of these, only one-third required further intervention with hysterectomy, resulting in a 67.8% success rate for expectant management [11]. These results highlight that expectant management has a role in managing PAS, and further studies are needed to clarify the conditions and severity of PAS cases that are suitable for this approach. By gaining insight into the different management strategies for varying degrees of placenta accreta, we can better tailor care for patients and improve their chances of uterine preservation. In this case, minimal placenta accreta was identified intraoperatively, with approximately 5% remaining adherent after initial removal attempts. The decision was made to pursue uterine preservation. At the six-week postpartum visit, suspected remnants of the placenta were found in the uterine cavity. Uterine artery embolization or hysterectomy would have been considered if hysteroscopic resection of the retained placenta had been unsuccessful. During the office visit, the patient expressed that she did not desire future fertility and would have preferred a hysterectomy if hysteroscopic resection had failed. After discussing hysterectomy and conservative options, the patient ultimately decided to pursue hysteroscopic removal and dilation, and curettage. The Mirena IUD was also placed to reduce bleeding and promote endometrial thinning. This combination successfully preserved this patient’s uterus and may offer a viable option for similar patients, though larger studies are needed to evaluate its efficacy in partial accreta management.

## Conclusions

The management of PAS continues to evolve, with research investigating various conservative management strategies compared to traditional approaches like cesarean hysterectomy. Intraoperative discovery of placenta accreta often requires physicians to make time-sensitive decisions for the most appropriate management. Variations in the degree and severity of retained placenta can significantly influence these decisions. There is also variation in physicians’ comfort level and competency when managing PAS, which can influence clinical decision-making and outcomes. In our case, successful management of a partial accreta was achieved through a conservative sequential approach. This presents a promising alternative for uterine preservation in appropriate cases of partial placenta accreta. Further large-scale research is needed to refine conservative management strategies for partial placenta accreta.
